# Effect of healthy lifestyle index and lifestyle patterns on the risk of mortality: A community-based cohort study

**DOI:** 10.3389/fmed.2022.920760

**Published:** 2022-08-30

**Authors:** Peng Hu, Murui Zheng, Jun Huang, Huan-Ying Fan, Chun-Jiang Fan, Hui-Hong Ruan, Yue-Shuang Yuan, Wenjing Zhao, Harry H. X. Wang, Hai Deng, Xudong Liu

**Affiliations:** ^1^School of Public Health, Guangdong Pharmaceutical University, Guangzhou, China; ^2^Department of Epidemiology, School of Public Health, Sun Yat-sen University, Guangzhou, China; ^3^Guangzhou Center for Disease Control and Prevention, Guangzhou, China; ^4^Department of Geriatrics, Guangdong Provincial People’s Hospital, Institute of Geriatrics, Guangdong Academy of Medical Science, Guangzhou, China; ^5^Xinzao Hospital of Guangzhou Panyu District, Guangzhou, China; ^6^Community Health Service Center of Nancun Town, Guangzhou, China; ^7^Community Health Service Center of Hualong Town, Guangzhou, China; ^8^School of Public Health and Emergency Management, Southern University of Science and Technology, Shenzhen, China; ^9^JC School of Public Health and Primary Care, The Chinese University of Hong Kong, Hong Kong, Hong Kong SAR, China; ^10^Department of Cardiology, Guangdong Provincial People’s Hospital, Guangdong Cardiovascular Institute, Guangdong Academy of Medical Science, Guangzhou, China

**Keywords:** lifestyle, healthy lifestyle index, lifestyle pattern, mortality, cohort study

## Abstract

**Background:**

Limited evidence was available on the association of the integrated effect of multidimensional lifestyle factors with mortality among Chinese populations. This cohort study was to examine the effect of combined lifestyle factors on the risk of mortality by highlighting the number of healthy lifestyles and their overall effects.

**Methods:**

A total of 11,395 participants from the Guangzhou Heart Study (GZHS) were followed up until 1 January 2020. Individual causes of death were obtained from the platform of the National Death Registry of China. The healthy lifestyle index (HLI) was established from seven dimensions of lifestyle, and lifestyle patterns were extracted from eight dimensions of lifestyle using principal component analysis (PCA). Hazard ratios (HRs) and 95% confidence intervals (95% CIs) were estimated using the Cox proportional hazard regression model.

**Results:**

During 35,837 person-years of follow-up, 184 deaths (1.61%) were observed, including 64 from cardiovascular disease. After adjustment for confounders, HLI was associated with a 50% (HR: 0.50, 95% CI: 0.25–0.99) reduced risk of all-cause mortality when comparing the high (6–7 lifestyle factors) with low (0–2 lifestyle factors) categories. Three lifestyle patterns were defined and labeled as pattern I, II, and III. Lifestyle pattern II with higher factor loadings of non-smoking and low-level alcohol drinking was associated with a decreased risk of all-cause mortality (HR: 0.63, 95% CI: 0.43–0.92, *P*_–trend_ = 0.023) when comparing the high with low tertiles of pattern score, after adjustment for confounders. Every 1-unit increment of pattern II score was associated with a decreased risk (HR: 0.97, 95% CI: 0.95–0.99) of all-cause mortality. The other two patterns were not associated with all-cause mortality, and the association of cardiovascular mortality risk was observed with neither HLI nor any lifestyle pattern.

**Conclusion:**

The results suggest that the more dimensions of the healthy lifestyle the lower the risk of death, and adherence to the lifestyle pattern characterized with heavier loading of non-smoking and low-level alcohol drinking reduces the risk of all-cause mortality. The findings highlight the need to consider multi-dimensional lifestyles rather than one when developing health promotion strategies.

## Introduction

Non-communicable diseases (NCDs) have encroached on many low- and middle-income countries, and become the leading cause of death worldwide (1). Available evidence suggested that lifestyle factors were associated with multiple NCDs (2), and adherence to a healthy lifestyle was associated with a lower risk of NCDs and mortality (3–8). Meanwhile, multiple lifestyle risk factors may have a synergistic effect on adverse health outcomes (9, 10). Hence, reducing exposure to lifestyle risk factors is of great significance for public health prevention and medical resource allocation (11).

Many studies have constructed healthy lifestyle scores to reflect the combined impact of major lifestyle factors, including smoking, alcohol consumption, body mass index (BMI), unhealthy diet, and physical inactivity, on mortality (12–14). A systematic review and meta-analysis found that adherence to the healthiest lifestyles was associated with a 55 and 58% reduced risk of all-cause mortality and cardiovascular mortality, respectively, compared with the least-healthy lifestyles (15). However, other unmentioned factors may also play a significant role in mortality risk. For instance, sleep is a critical bodily function (16), and poor sleep quality has been identified as a risk factor for many adverse health outcomes, such as all-cause mortality, cardiovascular death, etc. (17–19). The mental state is also an important aspect of health, and people with mental disorders have a higher risk of death (20, 21). Pratt and colleagues found that people with anxiety and depression had significantly high mortality than people without such mental disorders (21). In addition, current research mainly reflected the overall impact of lifestyle through the number of lifestyle factors, while ignoring the possible interactions between each factor. Principal component analysis (PCA) is an effective approach to reflect the combined effect of different components by generating different patterns, and using such an approach can avoid ignoring the interaction between some components (22). The human lifestyle is a complex whole with multiple dimensions, and it is important to consider as many of these dimensions as possible when quantifying lifestyle and assessing its impact on health.

Therefore, this prospective cohort study aimed to examine the combined effect of major lifestyle factors, including leisure-time physical activity (LTPA), diet, BMI, smoking, alcohol drinking, sleep quality, and mental status (anxiety, and depression), on the risk of all-cause and cardiovascular mortality, by establishing a healthy lifestyle index (HLI) to reflect the number of healthy lifestyle components and generating the lifestyle patterns to reflect the whole lifestyle profile.

## Materials and methods

### Study population

Guangzhou Heart Study (GZHS) is an ongoing prospective population-based cohort study in South China. Details of GZHS can be seen in our previous reports (23–25). Briefly, a total of 12,013 permanent residents aged 35 years or more were enrolled using the multistage sampling method; the baseline survey was successfully conducted between July 2015 and August 2017. Participants included met the following criteria: permanent residents in Guangzhou, aged 35 years or older, and had lived in the selected communities for at least 6 months before the survey. Participants with incomplete information of covariates (19 participants) or with a history of cardiovascular diseases including atrial fibrillation, heart failure, myocardial infarction, and valvular heart disease (589 participants) were excluded. Finally, a total of 11,395 participants were available for further analysis.

This study was approved by the Guangzhou Medical Ethics Committee of the Chinese Medical Association and by the Ethical Review Committee for Biomedical Research, School of Public Health, Sun Yat-sen University. The study was performed in line with the Declaration of Helsinki and all participants provided informed consent.

### Outcome ascertainment

The outcome of interest was all-cause mortality and cardiovascular mortality. Individual causes of death up to 1 January 2020 were collected from China’s National Death Registry, Guangzhou Center for Disease Control and Prevention. The follow-up time was defined as the time from participation in the GZHS to the date of the decedent’s death or to the censoring date (1 January 2020) for survivors. The causes of death were coded by professional medical workers according to the 10th revision of the International Classification of Disease and were further classified as all-cause death and death from cardiovascular diseases (I00-I99).

### Assessment of lifestyle factors

Structured questionnaires were used to collect information on social demographics, lifestyle, and disease history by using face-to-face interviews. The social demographics included age (years), sex (male, female), education (<high school, high school, >high school), and marital status (married, others). The medical examination was performed on each participant; height and weight were measured to calculate BMI (kg/m^2^); a normal BMI was defined as BMI in the range of 18.5–23.9 kg/m^2^ according to the Chinese standard (26), otherwise as unhealthy BMI.

The exposure information on cigarette smoking and alcohol drinking was collected using a structured questionnaire. For cigarette smoking, participants who have never smoked or smoked < 100 cigarettes in their lifetime were classified as non-smokers, and those who have currently smoked or smoked ≥ 100 cigarettes in their lifetime were classified as smokers. For alcohol drinking, participants were asked to report their drinking status. Participants who reported “frequent drinking” were considered as high-level alcohol drinking, and those who reported “never drinking or alcohol cessation” or “occasional drinking” were considered as low-level alcohol drinking.

Dietary consumption from each participant was collected using a 22-item food frequency questionnaire (FFQ) (24). Participants were asked to report their intake frequency of each food item (<once per month, 1–3 times per month, 1–3 times per week, 4–6 times per week, and ≥ once per day) over the previous 12 months. A total of 12 major food items in FFQ (cereals, legumes, vegetables, fruit, dairy, nuts, fish and shrimps, poultry, red meat, fried foods, high-salt foods, and sugary beverages) were used to create a diet quality score based on the latest Chinese Dietary Guidelines (27). First, a point was assigned to each category of the intake frequency (Supplementary Table 1): for cereals, fruit, dairy, and nuts, 0, 2, 4, 6, and 8 points were assigned to < once a month, 1–3 times a month, 1–3 times a week, 4–6 times a week, and 1–6 times a day, respectively; for legumes, vegetables, fish and shrimps, and poultry, 0, 1, 2, 3, and 4 were assigned to < once a month, 1–3 times a month, 1–3 times a week, 4–6 times a week, and 1–6 times a day respectively; for red meat, fried food, high-salted food, and sugary beverages, 8, 6, 4, 2, and 0 points were assigned to < once a month, 1–3 times a month, 1–3 times a week, 4–6 times a week, and 1–6 times a day, respectively (6, 28). Then, the total score for the diet quality was equal to the sum of points of 12 selected food items. Accordingly, the diet quality score ranged from 0 (lowest) to 80 (highest). If the dietary quality score for a participant was 50 points or more (in the upper two-fifths of all participants), then this participant was considered to have a healthy diet, otherwise have an unhealthy diet.

LTPA was evaluated by a modified Global Physical Activity Questionnaire (29). The total volume of LTPA for each subject was calculated as the sum of volumes of eight categories of most common LTPA including Tai Chi/Qigong, housework, stroll, bicycling, brisk walking/exercises/Yangko, swimming, ball games (basketball, table tennis, badminton, etc.), long-distance running/aerobics dancing. The value of each LTPA was calculated by multiplying the duration of activity by its frequency and then by its intensity (quantified by the value of metabolic equivalent, MET). More details on the assessment of physical activity can be seen in our previous report (25). According to the guidelines on physical activity by World Health Organization (WHO), to attain substantial health benefits, adults should do at least 150–300 min of moderate-intensity aerobic physical activity, or at least 75–150 min of vigorous-intensity aerobic physical activity, or an equivalent combination of moderate- and vigorous-intensity activity throughout the week (30); this means that conducting activity with at least 10 MET-hours/week is suggested to reach the minimum level of the recommended standard (30).

Sleep quality was assessed with two questions (17). The participants were asked to answer the question “Did you feel tired after waking up in the morning?” If the response of a participant was “yes,” then the participant was further required to report the frequency of tiredness after waking up in the morning during the past year, with five choices of “every day,” “3–4 times per week,” “1–2 times per week,” “1–2 times per month,” and “never.” The participants were considered as having poor sleep quality if their choice were “every day” or “3–4 times per week”; otherwise, the participants were regarded as having good sleep quality.

Mental status including anxiety and depression were evaluated by the Self-Rating Anxiety Scale (SAS) and the Center for Epidemiologic Studies Depression Scale (CES-D) (31, 32). The SAS index score ranged from 25 to 100 and a participant with a SAS index score ≥ 45 was considered as having anxiety. The CES-D score ranges from 0 to 60 and a participant with ≥ 16 was considered as having depression. Because the number of participants with depression in our study was small, when establishing the HLI, the participants who had either anxiety neurosis or depression were classified as having an unhealthy mental status, otherwise as having a healthy mental status.

### Healthy lifestyle index establishment

The detail of the definition of the HLI was shown in Table 1. The HLI was established by using seven dominant factors including BMI, cigarette smoking, alcohol drinking, dietary quality, LTPA, sleep quality, and mental status. These factors were shown as dichotomous variables, and the definitions of these variables were mentioned above. The score for each lifestyle factor was defined as follows: BMI (1 = normal BMI, 0 = unhealthy BMI), alcohol drinking (1 = low-level alcohol drinking, 0 = high-level alcohol drinking), cigarette smoking (1 = non-smoker, 0 = smoker), diet quality (1 = healthy diet, 0 = unhealthy diet), LTPA (1 = reach the minimum level of the recommended standard by WHO, 0 = not reach the minimum level of the recommended standard by WHO), sleep quality (1 = good sleep quality, 0 = poor sleep quality), and mental status (1 = unhealthy mental status, 0 = healthy mental status). The total score for HLI was calculated as the sum of the scores of seven selected factors. The score for HLI ranged from zero to seven (healthiest) points. The HLI was further transformed to the categorical variable: low (0–2 score), moderate (3–5 score), and high (6–7 score).

**TABLE 1:** The definition of the healthy lifestyle index.

Variable	Points	Description
Diet quality		
	0	Unhealthy diet: dietary quality score < 50
	1	Healthy diet: dietary quality score ≥ 50
Body mass index (BMI)		
	0	Unhealthy BMI: overweight or obese (BMI < 18.5 kg/m^2^ or BMI ≥ 24 kg/m^2^)
	1	Normal BMI: normal weight (18.5 ≤ BMI < 24 kg/m^2^)
Cigarette smoking		
	0	Smoker: current smoker or former smoker (≥100 cigarettes)
	1	Non-smoker: never smoker or former smoker (<100 cigarettes)
Alcohol drinking		
	0	High-level: often drinking
	1	Low-level: no drinking or occasionally drinking
leisure-time physical activity (LTPA)		
	0	Unhealthy: did not achieve the minimum level of the recommended standard
	1	Healthy: achieved the minimum level of the recommended standard
Sleep quality		
	0	Unhealthy: Felt tired after waking up in the morning more than 1–2 times per week
	1	Healthy: felt tired after waking up in the morning less than 1–2 times per week
Mental status		
	0	Unhealthy mental status: either anxiety or depression
	1	Healthy mental status: neither anxiety nor depression
Lifestyle index		
	0–2	Low: score of lifestyle index ranged from 0 to 2
	3–5	Moderate: score of lifestyle index ranged from 3 to 5
	6–7	High: score of lifestyle index ranged from 6 to 7

### Lifestyle pattern extraction

The lifestyle pattern was extracted by using the PCA with the varimax-rotated transformation from eight lifestyle components: BMI, diet quality, LTPA, depression (CES-D score), anxiety (SAS index score), cigarette smoking, alcohol drinking, and sleep quality. To perform the PCA, the categorical variables of cigarette smoking, alcohol drinking, and sleep quality were transformed into the continuous variable. For cigarette smoking, a score of 1 and 0 was assigned to the non-smoker and the smoker, respectively; for alcohol drinking, a score of 1 and 0 was assigned to the low-level alcohol drinking and the high-level alcohol drinking, respectively; for sleep quality, a simple score was assigned to each category of the frequency of tiredness after waking up in the morning during the past year: 1 = “every day,” 2 = “3–4 times per week,” 4 = “1–2 times per week,” 5 = “never.”

PCA used the correlation matrix of different lifestyle factors to identify common patterns of lifestyle factors within the data to account for the largest amount of variation in lifestyle. The varimax rotation is a statistical technique used to evaluate the relationship among individual factors (22). The Kaiser-Meyer-Olkin (KMO) criterion and Bartlett’s test of sphericity were used to evaluate the suitability of using the data for PCA (22). A positive loading for a lifestyle component indicated a direct association with the pattern, while a negative loading showed that this lifestyle component inversely contributed to the pattern. The components in each lifestyle pattern with absolute rotated factor loadings of ≥ 0.40 were referred to as “dominant components” hereafter.

Three lifestyle patterns were finally extracted and labeled as pattern I, pattern II, and pattern III (Supplementary Table 2). The pattern I was characterized with a higher loading of sleep quality, anxiety, and depression, explaining about 37.7% of total variance; Pattern II was characterized with a higher loading of low-level alcohol drinking and non-smoker, explaining about 32.8% of the total variance; Pattern III was characterized with a higher loading of LTPA, BMI, and diet quality, explaining about 29.5% of the total variance. The score of each pattern was calculated with the weighted approach by using rotated loadings as the weight (33): pattern score = $\sum_{1}^{21}v\,a\,r\,i\,a\,b\,l\,e_{i}\,w\,e\,i\,g\,h\,t_{i}$; ***variable*** represents each item; ***weight*** means the factor loading. Then the score of each pattern was transformed to categorical variables by using the tertile method.

### Statistical analysis

The distribution of baseline social-demographics, lifestyle factors, and other covariables was depicted and the difference among different groups was examined by chi-square test for a categorical variable and *t*-test, or Wilcoxon rank-sum test, or Kruskal-Wallis rank-sum test, or one-way analysis of variance for a continuous variable.

Cox proportional hazard regression model was performed to estimate the hazard ratio (HR) and their 95% confidence interval (Cl) before and after adjustment for confounders, to demonstrate the effects of individual lifestyle factors, HLI, and three lifestyle patterns on all-cause mortality and cardiovascular mortality. The proportional hazard assumption was examined using the Schoenfeld residuals method, and no significant violation of the assumption was observed. The linear exposure-response trend was examined by using Wald statistic.

Two sensitivity analyses were conducted to test the robustness of the results. To eliminate the possible effect of weight loss secondary to preclinical diseases, we excluded the participants with a BMI below 18.5 kg/m^2^. To exclude the possibility of reverse causality, we excluded participants who died within the first year after they finished the baseline survey. A product term between sex and HLI or each lifestyle pattern was added in the model to evaluate the possible multiplicative interaction by using the likelihood ratio test; however, no significant interaction was observed. All analyses were performed with R 4.0.1 (R Development Core Team, Vienna, Austria); the tests were two-tailed, and a *P*-value of less than 0.05 was considered statistically significant.

## Results

During 35,837 person-years of follow-up, 184 deaths (1.61%) were observed, including 64 deaths from cardiovascular disease. As shown in Table 2, of all subjects, the mean (*SD*) of age, BMI, diet quality, CES-D score, and SAS index score was 58.36 (11.70) years, 23.97 (3.53) kg/m^2^, 48.78 (7.55), 11.95 (2.42), and 43.91 (3.16), respectively. The median (IQR) value of LTPA was 34.65 (41.50) MET-h/week. Most participants were females (65.41%), with an education less than high school (63.0%), married (85.27%), non-smokers (79.61%), and never or occasion drinkers (94.01%).

**TABLE 2:** The characteristics of participants.

	Total (*N* = 11,395)	Lived (*N* = 11,211)	Death (*N* = 184)	*P-value*
Age, years, mean (*SD*)	58.36 (11.70)	58.16 (11.60)	70.84 (10.98)	< 0.001*
BMI, kg/m^2^, mean (*SD*)	23.97 (3.53)	23.98 (3.53)	23.34 (3.81)	0.026*
LTPA, MET-h, median (interquartile)	34.65 (41.50)	34.65 (41.33)	24.50 (34.91)	< 0.001^†^
Diet quality score, mean (*SD*)	48.78 (7.55)	48.79 (7.53)	48.02 (8.52)	0.223*
CES-D score, mean (*SD*)	11.95 (2.42)	11.95 (2.41)	12.00 (3.05)	0.839*
SAS index, mean (*SD*)	43.91 (3.16)	43.91 (3.16)	43.85 (3.33)	0.811*
**Sex, *N* (%)**				< 0.001^‡^
Male	3,941 (34.59)	3,843 (34.28)	98 (53.26)	
Female	7,454 (65.41)	7,368 (65.72)	86 (46.74)	
**Education, *N* (%)**				0.002^‡^
< High school	7,179 (63.00)	7,041 (62.80)	138 (75.00)	
High school	2,750 (24.13)	2,723 (24.29)	27 (14.67)	
>High school	1,466 (12.87)	1,447 (12.91)	19 (10.33)	
**Material status, *N* (%)**				< 0.001^‡^
Married	9,716 (85.27)	9,587 (85.51)	129 (70.11)	
Others	1,679 (14.73)	1,624 (14.49)	55 (29.89)	
**Diet quality, *N* (%)**				0.438^‡^
Unhealthy	6,212 (54.52)	6,106 (54.46)	106 (57.61)	
Healthy	5,183 (45.48)	5,105 (45.54)	78 (42.39)	
**Cigarette smoking, *N* (%)**				< 0.001^‡^
Smoker	2,324 (20.39)	2,265 (20.20)	59 (32.07)	
Non-smoker	9,071 (79.61)	8,946 (79.80)	125 (67.93)	
**Alcohol drinking, *N* (%)**				0.068^‡^
Low-level	10,713 (94.01)	10,540 (94.01)	173 (94.02)	
High-level	682 (5.99)	671 (5.99)	11 (5.98)	
**Mental health, *N* (%)**				0.814^‡^
Unhealthy	4,275 (37.52)	4,208 (37.53)	67 (36.41)	
Healthy	7,120 (62.48)	7,003 (62.47)	117 (63.59)	
**BMI, *N* (%)**				0.888^‡^
Unhealthy	5,882 (51.62)	5,792 (51.66)	90 (48.91)	
Normal	5,513 (48.38)	5,419 (48.34)	94 (51.09)	
**Sleep quality, *N* (%)**				0.214^‡^
Unhealthy	1,762 (15.46)	1,727 (15.40)	35 (19.02)	
Healthy	9,633 (84.54)	9,484 (84.60)	149 (80.98)	
**LTPA, *N* (%)**				0.255^‡^
Unhealthy	1,677 (14.72)	1,644 (14.66)	33 (17.93)	
Healthy	9,718 (85.28)	9,567 (85.34)	151 (82.07)	

BMI, body mass index; LTPA, leisure-time physical activity; MET-h, metabolic equivalent values-hours.

*P-values of continuous variables were from t-test.

^†^*P*-values of leisure-time physical activity was from Wilcoxon rank sum test.

^‡^*P*-values of categorical variables were from chi-square tests.

Individuals with higher HLI were more likely to be female and married (Supplementary Table 3). The proportion of participants with the higher education level increased with the increase of the HLI. With the increase of the HLI, participants had a higher level of healthy diet quality, LTPA, sleep quality, and mental health, and a higher proportion of normal BMI, non-smoking, and non-drinking. The means of lifestyle pattern II score and pattern III score were significantly higher in the lived group (*P* < 0.05), while both lived and death groups had a similar score of lifestyle pattern I (*P* > 0.05) (Supplementary Table 4).

As shown in Table 3, participants with good sleep quality were associated with a 38% (HR: 0.62, 95% CI: 0.43–0.91) reduced risk of all-cause mortality after adjustment for confounders. No significant association was observed between sleep quality and cardiovascular mortality risk. Other individual factors were not observed to be associated with the risk of all-cause and cardiovascular mortality.

**TABLE 3:** Association of individual lifestyle factors with mortality.

	All-cause mortality			Cardiovascular mortality		
		
	*N* (person-years/death)	Crude HR (95% CI)*	Adjusted HR (95% CI)^†^	*N* (person-years/death)	Crude HR (95% CI)*	Adjusted HR (95% CI)^†^
**Diet quality**						
Unhealthy	19,353/106	1.00	1.00	19,353/38	1.00	1.00
Healthy	16,484/78	0.86 (0.64, 1.15)	0.86 (0.63, 1.17)	16,484/26	0.79 (0.48, 1.31)	0.82 (0.49, 1.38)
**BMI**						
Unhealthy	18,424/90	1.00	1.00	18,424/35	1.00	1.00
Normal	17,413/94	1.10 (0.82, 1.47)	1.11 (0.83, 1.48)	17,413/29	0.87 (0.53, 1.43)	0.90 (0.55, 1.47)
**Cigarette smoking**						
Smoker	7,290/59	1.00	1.00	7,290/18	1.00	1.00
Non-smoker	28,548/125	0.54 (0.40, 0.74)	0.91 (0.62, 1.32)	28,548/46	0.65 (0.38, 1.12)	0.78 (0.40, 1.54)
**Alcohol drinking**						
Low-level	2,128/11	1.00	1.00	2,128/1	1.00	1.00
High-level	33,709/173	0.99 (0.54, 1.82)	1.50 (0.80, 2.80)	3,309/63	3.97 (0.55, 28.59)	5.23 (0.71, 38.43)
**LTPA**						
Unhealthy	5,232/33	1.00	1.00	5,232/14	1.00	1.00
Healthy	30,606/151	0.78 (0.54, 1.14)	0.88 (0.60, 1.29)	30,606/50	0.61 (0.34, 1.10)	0.67 (0.37, 1.23)
**Sleep quality**						
Unhealthy	5,541/35	1.00	1.00	5,541/9	1.00	1.00
Healthy	30,296/149	0.78 (0.54, 1.13)	0.62 (0.43, 0.91)	30,296/55	1.12 (0.55, 2.26)	0.92 (0.45, 1.89)
**Mental health**						
Unhealthy	13,297/67	1.00	1.00	13,297/22	1.00	1.00
Healthy	22,541/117	1.02 (0.76, 1.38)	1.11 (0.82, 1.51)	22,541/42	1.12 (0.67, 1.87)	1.24 (0.73, 2.09)

BMI, body mass index; CI, confidence interval; CVD, cardiovascular disease; HR, hazard ratio; LTPA, leisure-time physical activity; MET-h, metabolic equivalent values-hours.

*Adjustment for age, sex, education, and marital status.

^†^Adjustment for age, sex, education, marital status, and other lifestyle factors.

Table 4 shows the association of HLI with mortality risk. In comparison to the subjects within the low category of the HLI, those within both the moderate category (HR: 0.48, 95% CI: 0.25–0.93) and the high category (HR: 0.50, 95% CI: 0.25–0.99) were associated with a reduced risk of all-cause mortality after adjustment for confounders. However, no significant association was observed between HLI and cardiovascular mortality risk.

**TABLE 4:** Associations of healthy lifestyle index with mortality.

	*N* (person-years/death)*	Crude HR (95% CI)^†^	Adjusted HR (95% CI)^‡^
**All-cause mortality**			
Low (0–2 score)	928/10	1.00	1.00
Moderate (3–5 score)	21,460/113	0.49 (0.25, 0.93)	0.48 (0.25, 0.93)
High (6–7 score)	13,449/61	0.42 (0.21, 0.81)	0.50 (0.25, 0.99)
Moderate and high (3–7 score)	34,909/174	0.45 (0.24, 0.87)	0.49 (0.25, 0.93)
*P* for trend		0.013	0.124
Every 1-score increment		0.88 (0.79, 0.99)	0.95 (0.84, 1.07)
**Cardiovascular mortality**			
Low (0–2 score)	928/3	1.00	1.00
Moderate (3–5 score)	21,460/40	0.57 (0.18, 1.85)	0.48 (0.15, 1.57)
High (6–7 score)	13,449/21	0.47 (0.14, 1.59)	0.47 (0.14, 1.65)
Moderate and high (3–7 score)	34,909/61	0.53 (0.17, 1.70)	0.48 (0.15, 1.55)
*P* for trend		0.205	0.344
Every 1-score increment		0.90 (0.74, 1.10)	0.94 (0.76, 1.17)

*N represents the sample size.

^†^Crude HR, without any adjustment.

^‡^Adjusted HR, adjustment for age, sex, marital status, educational status.

When comparing the highest with lowest tertiles of pattern score, lifestyle pattern II was associated with a 37% (HR: 0.63, 95% CI: 0.43–0.92, *P*_–trend_ = 0.023) reduced risk of all-cause mortality after adjustment for confounders (Table 5). Every 1 score increment of the lifestyle pattern II was associated with a 3% (HR: 0.97, 95% CI: 0.95–0.99) reduced risk of all-cause mortality. No significant association was observed between the other two lifestyle patterns and the risk of all-cause mortality, and between three lifestyle patterns and the risk of cardiovascular mortality.

**TABLE 5:** The association of lifestyle patterns with mortality.

Patterns*	*N* (person-years/death)*	Crude HR (95% CI)^†^	Adjusted HR (95% CI)^‡^
**All-cause mortality**			
**Pattern I**			
Tertile 1	12,272/66	1.00	1.00
Tertile 2	11,800/62	0.99 (0.70, 1.40)	1.02 (0.72, 1.45)
Tertile 3	11,766/56	0.90 (0.63, 1.28)	0.91 (0.64, 1.30)
P for trend		0.568	0.621
Every 1-score increment		1.01 (0.97, 1.04)	1.01 (0.97, 1.04)
**Pattern II**			
Tertile 1	11,860/83	1.00	1.00
Tertile 2	11,939/57	0.68 (0.49, 0.95)	0.76 (0.54, 1.07)
Tertile 3	12,039/44	0.52 (0.36, 0.75)	0.63 (0.43, 0.92)
P for trend		<0.001	0.023
Every 1-score increment		0.96 (0.94, 0.98)	0.97 (0.95, 0.99)
**Pattern III**			
Tertile 1	11,812/74	1.00	1.00
Tertile 2	11,952/64	0.85 (0.61, 1.19)	0.90 (0.64, 1.27)
Tertile 3	12,073/46	0.60 (0.42, 0.87)	0.70 (0.48, 1.02)
P for trend		0.007	0.065
Every 1-score increment		0.99 (0.98, 0.99)	0.99 (0.98, 1.00)
**Cardiovascular mortality**			
**Pattern I**			
Tertile 1	12,272/26	1.00	1.00
Tertile 2	11,800/21	0.86 (0.48, 1.52)	0.89 (0.50, 1.58)
Tertile 3	11,766/17	0.69 (0.38, 1.28)	0.67 (0.36, 1.23)
P for trend		0.818	0.555
Every 1-score increment		1.01 (0.95, 1.07)	1.00 (0.94, 1.06)
**Pattern II**			
Tertile 1	11,860/30	1.00	1.00
Tertile 2	11,939/20	0.66 (0.38, 1.17)	0.74 (0.42, 1.31)
Tertile 3	12,039/14	0.46 (0.24, 0.86)	0.54 (0.28, 1.04)
P for trend		0.015	0.063
Every 1-score increment		0.97 (0.94, 0.99)	0.97 (0.94, 1.00)
**Pattern III**			
Tertile 1	11,812/26	1.00	1.00
Tertile 2	11,952/25	0.91 (0.53, 1.57)	0.99 (0.57, 1.71)
Tertile 3	12,074/13	0.43 (0.22, 0.85)	0.49 (0.25, 1.00)
P for trend		0.015	0.050
Every 1-score increment		0.98 (0.97, 0.99)	0.98 (0.97, 1.01)

*N represents the sample size. The pattern I was characterized with a higher loading of sleep quality, anxiety, and depression; Pattern II was characterized with a higher loading of low-level alcohol drinking and non-smoker; Pattern III was characterized with a higher loading of LTPA, BMI, and diet quality.

^†^Crude HR, without any adjustment.

^‡^Adjusted HR, adjustment for age, sex, marital status, educational status.

Two sensitivity analyses were conducted by excluding participants with a BMI lower than 18.5 kg/m2 and by excluding participants who died within the first year during the follow-up. Similar results as the main analyses were obtained for HLI (Supplementary Tables 5, 6) and three lifestyle patterns (Supplementary Tables 7, 8).

## Discussion

In this large prospective cohort study, we found that both moderate and high levels of HLI were associated with the reduced risk of all-cause mortality, and lifestyle pattern II characterized with higher loadings of low-level alcohol drinking and non-smoker was negatively associated with the risk of all-cause mortality, after adjustment for confounders.

The HLI established in this study considered many aspects of the lifestyle as comprehensively as possible. The five factors used in this study—LTPA, cigarette smoking, alcohol drinking, diet quality, and BMI—have also been considered in many other similar studies (7, 14, 34). In addition, this study also considered sleep quality and mental status, as they were important elements affecting the occurrence and death of chronic diseases (20, 35–38). Consistently, other studies on the combined or overall effects of lifestyle factors had also highlighted the contribution of sleep condition (9, 10, 13, 39) and mental status (9, 10). Our study found that both moderate level (3–5 score) and high level (6–7 score) of HLI decreased the risk of all-cause mortality, which was consistent with many other studies (13, 34, 39). However, the trend test was not reaching significance; this might be due to the limited death cases during a relatively short-time follow-up.

The multicollinearity and potential synergy were often observed among different individual lifestyle factors. The cumulative impact of multiple lifestyle components in a lifestyle pattern may be detectable. PCA is a multivariate technique that evaluates intercorrelations between individual habits or behaviors and has been widely applied in health science (22). In our study, three lifestyle patterns were successfully extracted by using PCA from eight components of non-smoking, low-level alcohol drinking, BMI, LTPA, diet quality, sleep quality, the SAS index score, and the CES-D score. We found that lifestyle pattern II which was characterized with higher factor loadings of non-smoking and low-level alcohol drinking was inversely associated with the risk of all-cause mortality, no matter the pattern score was regarded as a continuous variable or as a categorical variable. Similarly, Navarro and colleagues used PCA to extract dietary and lifestyle patterns and found that the pattern with the loadings most heavily on alcohol and cigarette use was associated with an increased risk of esophageal cancer (40); Al Thani et al. found that the pattern characterized by smoking, fast foods, sweetened beverages, and sweets was positively related to the risk of elevated blood pressure (41).

Non-smoking and low-level alcohol drinking are two dominant components of lifestyle pattern II. Literature agreed that cigarette smoking was the risk factor of premature mortality and various diseases including respiratory diseases, CVDs, diabetes, and cancer (42–45). Underlying mechanisms were that burning tobacco cigarettes can produce many chemicals that affect many aspects of human health, such as nicotine, nitrosamines, and polycyclic aromatic hydrocarbons. Nicotine affects cardiac contractility and heart rate, increases blood pressure, reduces sensitivity to insulin, aggravates diabetes, and results in endothelial dysfunction (46). Nitrosamines and polycyclic aromatic hydrocarbons are proven as carcinogens and CVD enhancers (47). Regarding low-level alcohol drinking, the Global Burden of Disease Study concluded that zero standard drinks per week minimized the overall health risk (48), namely no level of alcohol consumption improves health (49).

Noteworthy, our results found that high sleep quality was associated with a 38% reduced risk of all-cause mortality, which was consistent with previous studies (17–19). The underlying biological mechanism is that poor sleep quality may induce inflammatory cytokines (50), and inflammation has been associated with the incidence of cancer and CVDs (51, 52). No significant associations of diet quality, BMI, cigarette smoking, alcohol drinking, LTPA, and mental status with mortality were observed in this study. However, we found that combined healthy lifestyle factors decreased the risk of all-cause mortality. The possible reason for such discrepancies may be that when multiple lifestyle factors were combined, the synergistic effect of various components might be greater than the effect of each component (9). This further demonstrates the critical importance of considering a variety of healthy lifestyles when maintaining and promoting health.

This study has several strengths. First, participants from community-dwelling residents were recruited by using the multistage sampling method, which can to some degree minimize or reduce selection bias and ensure the population had better representativeness. Moreover, the questionnaire survey was conducted face to face by strictly trained investigators, which can reduce the information bias to a large degree. Second, we considered a relatively comprehensive range of lifestyle factors, including traditional and emerging lifestyle factors, which could reflect to some extent the complexity of modern life. Third, using indicators of the HLI and lifestyle patterns, we for the first time comprehensively examined the impact of lifestyles on the death from both aspects of the number of healthy lifestyles and their overall effects.

Some limitations also existed. First, the follow-up period in our study was relatively short, resulting in a limited number of death cases; thus, some results did not have enough power to detect significant findings. For example, we found that the association of HLI with cardiovascular mortality risk did not a reach significant level, contradicting the previous reports that showed a negative association between healthy lifestyles and cardiovascular mortality. However, the GZHS is an ongoing cohort study, and further follow-up studies will be performed to examine the association of lifestyle with the risk of cause-specific mortality. Second, dietary information over the past 12 months was collected using the FFQ, which might inevitably lead to recall bias. However, physical examination and questionnaire survey were conducted by trained investigators, which can reduce the bias to some degree. Third, although this study adjusted for several possible confounders, we cannot avoid the possibility of residual confounding due to unmeasured factors.

## Conclusion

Our results suggested that the more dimensions of the healthy lifestyle the lower the risk of death, and adherence to the lifestyle pattern characterized with heavier loading of non-smoking and lower alcohol drinking reduces the risk of all-cause mortality. The findings highlight the need to consider multi-dimensional lifestyles rather than one when developing health promotion strategies.

## Data availability statement

The raw data supporting the conclusions of this article will be made available by the authors, without undue reservation.

## Ethics statement

The studies involving human participants were reviewed and approved by the Guangzhou Medical Ethics Committee of the Chinese Medical Association and the Ethical Review Committee for Biomedical Research, School of Public Health, Sun Yat-sen University. The patients/participants provided their written informed consent to participate in this study.

## Author contributions

XL conceived and designed the study. MZ collected the mortality data. HD, MZ, JH, H-YF, C-JF, H-HR, and Y-SY collected all other data. PH analyzed the data. PH, MZ, and JH drafted the manuscript. HD, XL, WZ, and HW reviewed and edited the manuscript. All authors provided comments and approved the final version.
