# Supplementary material for: Effect of healthy lifestyle index and lifestyle patterns on the risk of mortality: A community-based cohort study
Source: Front Med (Lausanne). 2022 Aug 30;9:920760. doi: 10.3389/fmed.2022.920760 (PMC9468322; doi:10.3389/fmed.2022.920760)
Supplement: Supplementary file 1 [file Table_1.pdf]

Supplementary Table S1. Components of the dietary quality and scores for intake frequency of each food item

Food items	Food frequency questionnaire responses				
	< once per month	1-3 times per month	1-3 times per week	4-6 times per week	≥ once per week
Cereals	0	2	4	6	8
Fruit	0	2	4	6	8
Dairy	0	2	4	6	8
Nuts	0	2	4	6	8
Legumes	0	1	2	3	4
Vegetables	0	1	2	3	4
Fish and shrimps	0	1	2	3	4
poultry	0	1	2	3	4
Red meat	8	6	4	2	0
Fried foods	8	6	4	2	0
High-salt foods	8	6	4	2	0
Sugary beverage	8	6	4	2	0

Supplementary Table S2. Rotated factor loadings for each lifestyle pattern

Items *	Pattern I	Pattern II	Pattern III	Communality	Uniqueness	Complexity
LTPA	0.004	0.294	0.524	0.355	0.645	1.543
BMI	-0.016	0.343	-0.667	0.562	0.438	1.495
Diet quality	-0.052	0.219	0.657	0.482	0.518	1.233
Non-smoker	0.037	0.744	0.073	0.591	0.409	1.133
Low-level alcohol drinking	0.004	0.732	0.189	0.536	0.464	1.000
Sleep quality	-0.401	-0.010	0.011	0.166	0.834	1.068
SAS index score	0.824	0.027	0.010	0.680	0.320	1.002
CES-D score	0.831	-0.002	0.094	0.699	0.301	1.025
SS loadings	1.535	1.337	1.200			
Proportion Explained	0.377	0.328	0.295			
Cumulative Proportion	0.377	0.705	1.000			

Abbreviations: BMI, body mass index; LTPA, leisure-time physical activity.

* Lifestyle patterns were extracted by using principal component analysis. Mean item complexity was 1.2. Test of the hypothesis that 3 components were sufficient. The root mean square of the residuals (RMSR) was 0.14, with the empirical chi square of 12288 and with probability of less than 0.001. A positive loading for a lifestyle component indicated a direct association with the pattern, while a negative loading showed that this lifestyle component inversely contributed to the pattern. The score of each pattern was calculated by using weighted methods; pattern score = $\sum_{1}^{21} variable_i \times weight_i$; ***variable*** represents each item; ***weight*** means the factor loading.

Supplementary Table S3. Characteristic of participants by healthy lifestyle index

Characteristic	Total (N=11395)	Low (N=302)	Moderate (N=6877)	High (N=4216)	*P* value
Age, years, mean (S.D.)	58.36 (11.70)	56.30 (10.93)	58.53 (11.73)	58.23 (11.70)	0.939*
BMI, kg/m^2, mean (S.D.)	23.97 (3.53)	25.30 (3.78)	24.65 (3.71)	22.75 (2.81)	<0.001*
LTPA, MET-h, median (Interquartile)	34.65 (41.5)	3.50 (11.20)	29.30 (40.95)	44.80 (43.08)	<0.001†
Diet quality score, mean (S.D.)	48.78 (7.55)	42.42 (6.07)	46.52 (6.93)	52.92 (6.69)	<0.001*
CES-D score, mean (S.D.)	11.95 (2.42)	12.77 (3.45)	12.10 (2.58)	11.66 (1.99)	<0.001*
SAS index, mean (S.D.)	43.91 (3.16)	46.01 (3.13)	44.41 (3.34)	42.94 (2.53)	<0.001*
Sex, N (%)					<0.001‡
Male	3941 (34.59)	272 (90.07)	2828 (41.12)	841 (19.95)	
Female	7454 (65.41)	30 (9.93)	4049 (58.88)	3375 (80.05)	
Education, N (%)					<0.001‡
< high school	7179 (63.00)	211 (69.87)	4678 (68.02)	2290 (54.32)	
High school	2750 (24.13)	69 (22.85)	1471 (21.39)	1210 (28.70)	
> high school	1466 (12.87)	22 (7.28)	728 (10.59)	716 (16.98)	
Material status, N (%)					0.025‡
Married	9716 (85.27)	274 (90.73)	5855 (85.14)	3587 (85.08)	
Others	1679 (14.73)	28 (9.27)	1022 (14.86)	629 (14.92)	
Diet quality, N (%)					<0.001‡
Unhealthy	6212 (54.52)	280 (92.72)	4971 (72.28)	961 (22.79)	
Healthy	5183 (45.48)	22 (7.28)	1906 (27.72)	3255 (77.21)	
Cigarette smoking, N (%)					<0.001‡
Smoker	2324 (20.39)	260 (86.09)	1900 (27.63)	164 (3.89)	
Non-smoker	9071 (79.61)	42 (13.91)	5977 (72.37)	4052 (96.11)	
Alcohol drinking, N (%)					<0.001‡
Low-level	10713 (94.01)	146 (48.34)	6378 (92.74)	4189 (99.36)	
High-level	682 (5.99)	156 (51.66)	499 (7.26)	27 (0.64)	
Mental health, N (%)					<0.001‡
Unhealthy	4275 (37.52)	245 (81.13)	3399 (49.43)	631 (14.97)	
Healthy	7120 (62.48)	57 (18.87)	3478 (50.57)	3585 (85.03)	
BMI, N (%)					<0.001‡
Unhealthy	5882 (51.62)	257 (85.10)	4557 (66.26)	1068 (25.33)	
Normal	5513 (48.38)	45 (14.90)	2320 (33.74)	3148 (74.67)	
Sleep quality, N (%)					<0.001‡
Unhealthy	1762 (15.46)	152 (50.33)	1450 (21.08)	160 (3.80)	
Healthy	9633 (84.54)	150 (49.67)	5427 (78.92)	4056 (96.20)	
LTPA, N (%)					<0.001‡
Unhealthy	1677 (14.72)	222 (73.51)	1351 (19.65)	104 (2.47)	
Healthy	9718 (85.28)	80 (26.49)	5526 (80.35)	4112 (97.53)	

Abbreviations: BMI, body mass index; LTPA, leisure-time physical activity; MET-h, metabolic equivalent values-hours.

* *P* values of continuous variables were from one-way analysis of variance;

† *P* values of leisure-time physical activity was from kruskal-wallis rank sum test;

‡ *P* values of categorical variables were from chi-square tests.

Supplementary Table S4. Distribution of scores of three lifestyle patterns

Patterns*	Total	Lived	Death	*P* value
Score of Pattern I				
Mean (SD)	41.64 (4.08)	41.64 (4.07)	41.67 (4.83)	0.934
Range	27.5~99.3	27.5~99.3	32.7~70.8	
Score of Pattern II				
Mean (SD)	33.17 (10.47)	33.21 (10.49)	30.62 (8.82)	<0.001
Range	12.7~119	14.0~119	12.7~66.8	
Score of Pattern III				
Mean (SD)	39.93 (20.06)	40.00 (20.10)	35.99 (16.98)	0.007
Range	-1.73~196	-1.73~196	-0.27~103	

*The pattern I was characterized with a higher loading of sleep quality, anxiety, and depression; Pattern II was characterized with a higher loading of low-level alcohol drinking and non-smoker; Pattern III was characterized with a higher loading of LTPA, BMI, and diet quality. The score of each pattern was calculated by using weighted methods; pattern score = $\sum_1^{21} variable_i \times weight_i$; ***variable*** represents each item; ***weight*** means the factor loading.

Supplementary Table S5. Associations of combined healthy lifestyle index with mortality by excluding the participants who died within the first year of the follow-up

	N (person-years / death) *	Crude HR (95% CI) †	Adjusted HR (95% CI) ‡
All-cause mortality			
Low (0-2)	928 / 9	1.00	1.00
Moderate (3-5)	21450 / 93	0.44 (0.22, 0.88)	0.43 (0.21, 0.85)
High (6-7)	13442 / 50	0.37 (0.18, 0.76)	0.43 (0.21, 0.90)
P for trend		0.010	0.066
Every 1-score increment		0.88 (0.78, 0.99)	0.94 (0.82, 1.08)
CVD-specific mortality			
Low (0-2)	928 / 3	1.00	1.00
Moderate (3-5)	21450 / 32	0.45 (0.14, 1.49)	0.37 (0.11, 1.23)
High (6-7)	13442 / 18	0.40 (0.12, 1.36)	0.38 (0.11, 1.35)
P for trend		0.175	0.249
Every 1-score increment		0.90 (0.73, 1.12)	0.94 (0.74, 1.19)

Abbreviations: CI, confidence interval; CVD, cardiovascular disease; HR, hazard ratio.

*N represents the sample size.

† Crude HR, without any adjustment.

‡Adjusted HR, adjustment for age, sex, marital status, educational status.

Supplementary Table S6. Associations of combined healthy lifestyle index with mortality by excluding participants with a BMI below 18.5kg/m^2

	N (person-years / death) *	Crude HR (95% CI) †	Adjusted HR (95% CI) ‡
All-cause mortality			
Low (0-2)	853/10	1.00	1.00
Moderate (3-5)	20323/100	0.42 (0.22, 0.80)	0.41 (0.21, 0.79)
High (6-7)	13136/59	0.38 (0.19, 0.74)	0.43 (0.22, 0.87)
P for trend		0.011	0.068
Every 1-score increment		0.89 (0.79, 1.00)	0.94 (0.83, 1.07)
CVD-specific mortality			
Low (0-2)	853/3	1.00	1.00
Moderate (3-5)	20323/35	0.48 (0.15, 1.57)	0.41 (0.12, 1.36)
High (6-7)	13136/21	0.44 (0.13, 1.49)	0.44 (0.13, 1.52)
P for trend		0.239	0.353
Every 1-score increment		0.91 (0.74, 1.12)	0.94 (0.75, 1.18)

Abbreviations: CI, confidence interval; CVD, cardiovascular disease; HR, hazard ratio.

*N represents the sample size.

† Crude HR, without any adjustment.

‡Adjusted HR, adjustment for age, sex, marital status, educational status.

Supplementary Table S7. The association of lifestyle patterns with mortality by excluding participants who died within the first year

Patterns *	N (person-years / death) *	Crude HR (95% CI) †	Adjusted HR (95% CI) ‡
All-cause mortality			
Pattern I			
Tertile 1	12264 / 52	1.00	1.00
Tertile 2	11793 / 51	1.05 (0.71, 1.54)	1.08 (0.73, 1.59)
Tertile 3	11762 / 49	1.01 (0.68, 1.49)	1.02 (0.69, 1.50)
P for trend		0.953	0.913
Every 1-score increment		1.02 (0.98, 1.05)	1.02 (0.98, 1.06)
Pattern II			
Tertile 1	11851 / 68	1.00	1.00
Tertile 2	11952 / 48	0.70 (0.48, 1.01)	0.77 (0.53, 1.12)
Tertile 3	12016 / 36	0.52 (0.35, 0.78)	0.62 (0.41, 0.94)
P for trend		0.001	0.023
Every 1-score increment		0.97 (0.95, 0.99)	0.98 (0.96, 0.99)
Pattern III			
Tertile 1	11813 / 60	1.00	1.00
Tertile 2	11949 / 56	0.92 (0.64, 1.32)	0.97 (0.67, 1.40)
Tertile 3	12058 / 36	0.58 (0.38, 0.88)	0.66 (0.44, 1.01)
P for trend		0.009	0.056
Every 1-score increment		0.99 (0.98, 0.99)	0.99 (0.98, 0.99)
Cardiovascular mortality			
Pattern I			
Tertile 1	12264 / 22	1.00	1.00
Tertile 2	11793 / 16	0.78 (0.41, 1.49)	0.81 (0.42, 1.54)
Tertile 3	11762 / 15	0.73 (0.38, 1.41)	0.70 (0.36, 1.35)
P for trend		0.331	0.281
Every 1-score increment		1.01 (0.95, 1.08)	1.01 (0.95, 1.08)
Pattern II			
Tertile 1	11851 / 24	1.00	1.00
Tertile 2	11952 / 17	0.70 (0.38, 1.30)	0.77 (0.41, 1.45)
Tertile 3	12016 / 12	0.49 (0.24, 0.98)	0.56 (0.28, 1.15)
P for trend		0.042	0.113
Every 1-score increment		0.97 (0.94, 0.99)	0.97 (0.94, 1.01)
Pattern III			
Tertile 1	11813 / 21	1.00	1.00
Tertile 2	11949 / 22	1.03 (0.57, 1.87)	1.11 (0.61, 2.03)
Tertile 3	12058 / 10	0.46 (0.22, 0.98)	0.52 (0.24, 1.11)
P for trend		0.041	0.093
Every 1-score increment		0.98 (0.97, 0.99)	0.99 (0.97, 1.00)

*N represents the sample size. The pattern I was characterized with a higher loading of sleep quality, anxiety, and depression; Pattern II was characterized with a higher loading of low-level alcohol drinking and non-smoker; Pattern III was characterized with a higher loading of LTPA, BMI, and diet quality.

† Crude HR, without any adjustment.

‡ Adjusted HR, adjustment for age, sex, marital status, educational status.

Supplementary Table S8. The association of lifestyle patterns with mortality by excluding participants with a BMI below 18.5 kg/m^2

Patterns *	N (person-years / death) *	Crude HR (95% CI) †	Adjusted HR (95% CI) ‡
All-cause mortality			
Pattern I			
Tertile 1	11769 / 58	1.00	1.00
Tertile 2	11253 / 60	1.10 (0.77, 1.58)	1.15 (0.80, 1.65)
Tertile 3	11290 / 51	0.93 (0.64, 1.36)	0.94 (0.65, 1.38)
P for trend		0.757	0.816
Every 1-score increment		1.00 (0.97, 1.04)	1.01 (0.97, 1.05)
Pattern II			
Tertile 1	11358 / 72	1.00	1.00
Tertile 2	11445 / 57	0.78 (0.55, 1.11)	0.84 (0.59, 1.20)
Tertile 3	11510 / 40	0.55 (0.37, 0.80)	0.63 (0.42, 0.93)
P for trend		0.002	0.022
Every 1-score increment		0.97 (0.96, 0.99)	0.98 (0.96, 0.99)
Pattern III			
Tertile 1	11314 / 67	1.00	1.00
Tertile 2	11446 / 60	0.88 (0.62, 1.25)	0.93 (0.65, 1.32)
Tertile 3	11553 / 42	0.61 (0.41, 0.90)	0.69 (0.46, 1.02)
P for trend		0.011	0.061
Every 1-score increment		0.99 (0.98, 0.99)	0.99 (0.98, 0.99)
Cardiovascular mortality			
Pattern I			
Tertile 1	11769 / 22	1.00	1.00
Tertile 2	11253 / 20	0.97 (0.53, 1.78)	1.02 (0.56, 1.88)
Tertile 3	11290 / 17	0.82 (0.44, 1.55)	0.79 (0.42, 1.49)
P for trend		0.552	0.489
Every 1-score increment		1.02 (0.96, 1.08)	1.02 (0.96, 1.08)
Pattern II			
Tertile 1	11358 / 26	1.00	1.00
Tertile 2	11445 / 20	0.76 (0.43, 1.36)	0.82 (0.46, 1.49)
Tertile 3	11510 / 13	0.49 (0.25, 0.95)	0.57 (0.29, 1.12)
P for trend		0.035	0.100
Every 1-score increment		0.97 (0.94, 0.99)	0.97 (0.94, 1.01)
Pattern III			
Tertile 1	11314 / 25	1.00	1.00
Tertile 2	11446 / 23	0.90 (0.51, 1.59)	0.99 (0.56, 1.75)
Tertile 3	11553 / 11	0.43 (0.21, 0.87)	0.49 (0.24, 1.00)
P for trend		0.018	0.050
Every 1-score increment		0.98 (0.97, 0.99)	0.99 (0.97, 1.00)

*N represents the sample size. The pattern I was characterized with a higher loading of sleep quality, anxiety, and depression; Pattern II was characterized with a higher loading of low-level alcohol drinking and non-smoker; Pattern III was characterized with a higher loading of LTPA, BMI, and diet quality.

† Crude HR, without any adjustment.

‡ Adjusted HR, adjustment for age, sex, marital status, educational status.
